# Understanding the risk factors for adverse events during exchange transfusion in neonatal hyperbilirubinemia using explainable artificial intelligence

**DOI:** 10.1186/s12887-022-03615-5

**Published:** 2022-09-30

**Authors:** Shuzhen Zhu, Lianjuan Zhou, Yuqing Feng, Jihua Zhu, Qiang Shu, Haomin Li

**Affiliations:** The Children’s Hospital, Zhejiang University School of Medicine, National Clinical Research Center for Child Health, Hangzhou, China

**Keywords:** Exchange transfusion, Adverse events, Explainable artificial intelligence, Risk factors

## Abstract

**Objective:**

To understand the risk factors associated with adverse events during exchange transfusion (ET) in severe neonatal hyperbilirubinemia.

**Study design:**

We conducted a retrospective study of infants with hyperbilirubinemia who underwent ET within 30 days of birth from 2015 to 2020 in a children’s hospital. Both traditional statistical analysis and state-of-the-art explainable artificial intelligence (XAI) were used to identify the risk factors.

**Results:**

A total of 188 ET cases were included; 7 major adverse events, including hyperglycemia (86.2%), top-up transfusion after ET (50.5%), hypocalcemia (42.6%), hyponatremia (42.6%), thrombocytopenia (38.3%), metabolic acidosis (25.5%), and hypokalemia (25.5%), and their risk factors were identified. Some novel and interesting findings were identified by XAI.

**Conclusions:**

XAI not only achieved better performance in predicting adverse events during ET but also helped clinicians to more deeply understand nonlinear relationships and generate actionable knowledge for practice.

**Supplementary Information:**

The online version contains supplementary material available at 10.1186/s12887-022-03615-5.

## Introduction

Jaundice is a common condition in neonates. Approximately 60% and 80% of term and preterm infants, respectively, have clinical jaundice in the first week after birth, but only a very small proportion of them (0.02% and 0.16% in term or preterm infants, respectively) develop severe hyperbilirubinemia [1]. Severe neonatal hyperbilirubinemia can cause neurological disability, such as encephalopathy or mortality, if not effectively managed [2].

Phototherapy and exchange transfusion (ET) are the primary treatment modalities to prevent bilirubin encephalopathy [3, 4]. ET is a blood transfusion performed by removing blood and replacing it with blood from a donor. Although ET has become a rare event in most developed countries, it remains a frequent emergency rescue procedure for severe neonatal hyperbilirubinemia, especially in many developing countries [5, 6]. ET is effective and considered to be a safe procedure; however, it is not without risks, and the mortality rates range from 0.5% to 3.3% reported in a study [7]. Therefore, the current recommendations for performing ET are based on a balance between the risks of encephalopathy and the adverse events related to the procedure.

Common adverse events during ET include hyperglycemia, thrombocytopenia, hypocalcemia, hypokalemia or hyperkalemia, and metabolic acidosis, which can be monitored and corrected in a timely manner [8]. Although some risk factors for these adverse events have been reported in several studies [7–10], only risk factors with a linear relationship have been identified for these adverse events during ET using traditional data analysis. The potential benefits of novel artificial intelligence (AI) technologies applied in the clinic have been exciting and profound in recent years [11]. It also greatly affects neonate care [12]. The purpose of this study was to evaluate these adverse events during ET in neonatal hyperbilirubinemia and to identify the potential risk factors for these complications based on state-of-the-art explainable artificial intelligence (XAI) technology.

Although linear models have historically been popular because they are interpretable, modern complex machine learning models often achieve higher predictive accuracy because they capture complex interactions among variables, in addition to noting nonlinear relationships [13, 14]. In addition to the superior performance of modern machine learning models, some explainable artificial intelligence techniques, such as SHAP (SHapley Additive exPlanations), can better demonstrate nonlinear relationships (e.g., U-shaped relationships) [15, 16], and new relationships discovered by the model are even more valuable than the application of the model itself [17]. In particular, the discovery of new relationships can help medical professionals control some avoidable risks or prepare in advance for specific unavoidable risks.

## Subjects and methods

The medical records of neonates who received exchange transfusions to treat severe hyperbilirubinemia in neonatal units at the Children’s Hospital, Zhejiang University School of Medicine over a period of six years (from January 2015 through December 2020) were reviewed retrospectively. The indications for ET and the method of ET followed the relevant Chinese clinical guidelines [18], based on which the double volume exchange method (150–160 ml/kg) was completed for approximately 90–120 min. Blood gas, blood glucose, electrolytes, blood calcium, and blood cell counts were monitored during ET.

Patient characteristics, such as sex, gestational age, delivery mode, Apgar score at birth, weight at birth, weight at admission, age at admission, parents’ and baby’s blood group, mode of feeding before ET, the artery and vein used for ET, and relevant laboratory tests, such as direct bilirubin (DBIL), indirect bilirubin (IBIL) and total bilirubin (TBIL), serum calcium, glucose, sodium, potassium, white blood cells, hemoglobin, pH, and HCO_3_, were collected at different time points before, during and after ET.

Based on the WHO definition of adverse events: “an unexpected and undesired incident directly associated with the care or services provided to the patient” [19], adverse events during ET were defined by the following quantitative criteria that were outside the normal range for neonates. Hyperglycemia occurred when serum glucose was > 7.2 mmol/L, metabolic acidosis if HCO_3_ was < 18 mmol/L, hyperkalemia if serum potassium was ≥ 5.5 mmol/L, hypokalemia when serum potassium was < 3.0 mmol/L, hypocalcemia if serum calcium was < 0.9 mmol/L, thrombocytopenia if platelet count < 100 × 10^9^/L, hyponatremia if serum sodium was < 135 mmol/L, cyanosis if SpO_2_ < 90%, and top-up transfusion if the hemoglobin reduction met the clinical indications for transfusion. All indicators were monitored during the ET, following the clinical guidelines in China.

The neonates were categorized according to their status with/without specific adverse events during ET. Continuous variables (such as age and weight) of the patients were reported as the mean ± SD and were compared using the Mann‒Whitney U test. Categorical variables (such as sex) were reported as counts (percentages) and compared using the chi-square test. A *p* value < 0.05 was considered statistically significant.

Several widely used machine learning methods, such as Random Forest, XGBoost, logistic regression, Gaussian naïve Bayes, and K-neighbors, were used to train a machine learning model using 70% of the data and test them on the standby 30% of the data, which were split randomly. These five machine learning models were from the scikit-learn Python package. Random Forest and XGBoost are decision tree-based algorithms. Logistic regression is a widely used supervised learning algorithm that makes use of logistic functions to predict the probability of a binary outcome. Gaussian naïve Bayes is a classifier based on the Bayes theorem. K-neighbors is a nonparametric, supervised learning classifier that uses proximity to make classifications or predictions about the grouping of an individual data point.

In this study, the best performing model was enhanced with an interpretation method called SHAP [15], which is a game-theoretic approach for explaining the output of any machine learning model by computing each feature for the prediction. It calculates exact SHAP values for each feature. SHAP values are additive; they sum to the model’s output. They are also consistent, which means features that are unambiguously more important are guaranteed to have a higher SHAP value. Therefore, SHAP values are consistent and accurate calculations of each feature’s contribution to the model’s prediction. The SHAP for decision tree-based algorithms (such as Random Forest, XGBoost) called TreeExlainer also extends local explanations to capture pairwise interactions directly. In this study, higher SHAP values imply large contributions to adverse event risks. This explainable machine learning model helps clinicians understand the risk factors for a single prediction, for a single variable and for the entire dataset at different levels through visualization approaches. These explanations have the potential to generate human actionable knowledge to improve clinical outcomes.

## Results

There were 188 exchange transfusions performed in 185 neonates in this study, as shown in Table 1. Among them, 112 (59.6%) were male. Overall, 34 (18.1%) infants were preterm, and the mean gestational age was 37.93 ± 1.63 weeks old. The mean age at admission was 6.42 ± 3.97 days old, and exchange transfusion was performed 6.63 ± 3.40 h after admission. Among 188 cases, ABO incompatibility was found in 65 infants (34.6%), Rhesus (Rh) incompatibility in 21 (11.2%), G6PD deficiency in 30 (16.0%), and MN incompatibility, which is another antigen incompatibility that occurs very rarely, in 1 (0.5%). Bilirubin encephalopathy was diagnosed in 63 (33.5%) cases, sepsis was diagnosed in 22 (11.7%), anemia was diagnosed in 15 (8%), and NEC (necrotizing enterocolitis) and purulent meningitis were diagnosed in 2 (1.1%). Among all cases, 185 (98.4%) experienced different adverse events, and the most common adverse events were hyperglycemia (86.2%), followed by anemia requiring top-up transfusion after ET (50.5%), hypocalcemia (42.6%), hyponatremia (42.6%), thrombocytopenia (38.3%), acidosis (25.5%), hypokalemia (25.5%), hyperkalemia (3.2%), convulsions (2.7%) and cyanosis (2.7%). Considering the sample size, this study focused on only the 7 most common adverse events.

**Table 1:** Baseline demographic characteristics

Characteristic	Value
***Total ET count***	188
***Male sex***	112(59.6%)
***Gestational age (weeks)***	37.93 ± 1.63
***Premature***	34 (18.1%)
***Delivery by C-section***	66(35.1%)
***Apgar score***	9.88 ± 0.48
***Birth weight (g)***	3201.30 ± 429.75
***Weight at admission (g)***	3036.97 ± 430.53
***Age at admission (days)***	6.42 ± 3.97
***Length of stay***	9.31 ± 3.93
***Etiology***	
*Unidentified*	96(51.1%)
*ABO incompatibility*	65(34.6%)
*G6PD deficiency*	30(16.0%)
*Rh incompatibility*	21 (11.2%)
*MN incompatibility*	1 (0.5%)
***Other diagnosis***	
*Bilirubin encephalopathy*	63 (33.5%)
*Sepsis*	22(11.7%)
*Anemia*	15 (8%)
*Necrotizing enterocolitis*	2(1.1%)
*Purulent meningitis*	2(1.1%)
***Exchange transfusion (ET)***	
*ET volume (ml)*	495.27 ± 78.59
*ET time (minutes)*	96.77 ± 18.82
*TSB before ET*	388.70 ± 107.66
*TSB during ET*	253.41 ± 80.29
*TSB after ET*	198.75 ± 62.13
*TSB 1 day after ET*	220.60 ± 58.95
*Bilirubin exchange rate*	0.48 ± 0.10
***Adverse events during ET***	
*Hyperglycemia*	162(86.2%)
*Requiring top-up transfusion after ET*	95(50.5%)
*Hypocalcemia*	80(42.6%)
*Hyponatremia*	80(42.6%)
*Thrombocytopenia*	72(38.3%)
*Metabolic acidosis*	48(25.5%)
*Hypokalemia*	48(25.5%)
*Hyperkalemia*	6(3.2%)
*Convulsion*	5(2.7%)
*Cyanosis*	5(2.7%)

**Fig. 1:**
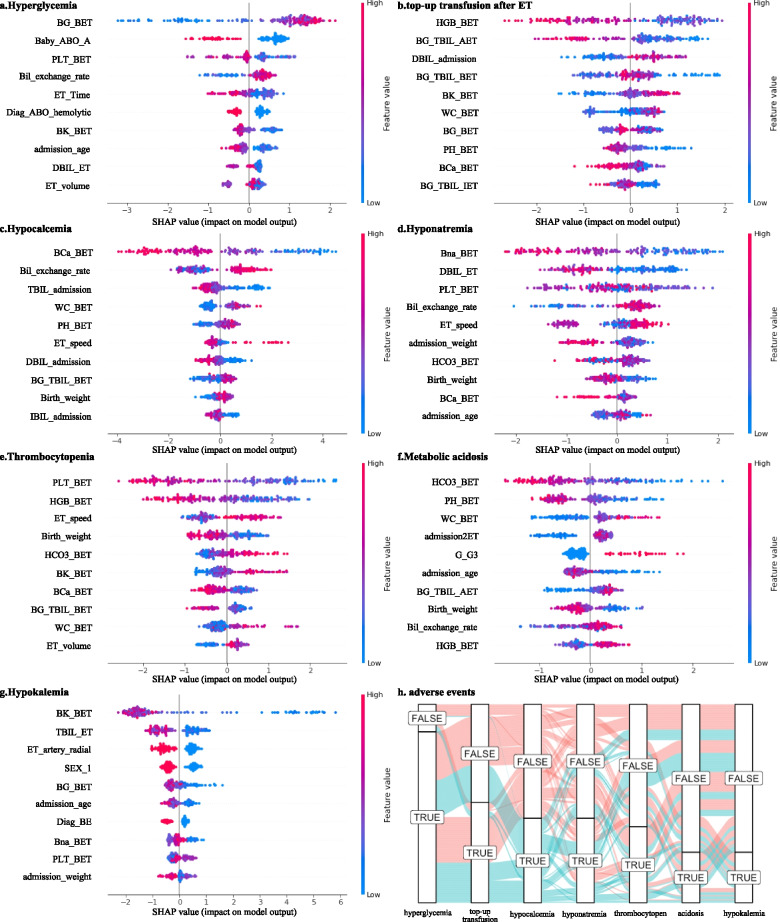
The top 10 most important variables identified by XAI for 7 adverse events during ET in neonatal hyperbilirubinemia. **a** Hyperglycemia; **b** top-up transfusion after ET; **c** hypocalcemia; **d** hyponatremia; **e** thrombocytopenia; **f** metabolic acidosis; **g** hypokalemia; **h** an alluvial diagram among these adverse events. The infants with/without hypocalcemia are represented in different colors

The variables with significant differences between patients with and without 7 common adverse events during ET are shown in Table 2. Please note that directly related variables regarding the adverse events, such as blood glucose for hyperglycemia and serum calcium for hypocalcemia, are not shown in this table. Younger age is a common risk factor for these adverse events, and there are significant differences in infants with/without thrombocytopenia, hypokalemia, top-up transfusion, and hypocalcemia. However, the term and preterm infants did not show significant differences in these adverse events. Female neonates are more likely to experience hypokalemia. Breastfeeding can reduce the risk of hypokalemia and hypocalcemia. Not starting feeding is a risk factor for metabolic acidosis and hypocalcemia. Formula feeding increases the risk of hypocalcemia. There was also no significant difference in the mode of delivery or Apgar score for adverse events during ET. Different etiologies of hyperbilirubinemia have different risks for different adverse reactions. ABO incompatibility is associated with a higher risk of metabolic acidosis but a lower risk of hyperglycemia and hyponatremia. RH incompatibility is associated with a higher risk of hypocalcemia. G6PD deficiency reduces the risk of hypocalcemia and hypokalemia. Etiology-identified neonates are more likely to experience top-up transfusion after ET. A short ET time contributes to hyperglycemia and hypocalcemia. The ET speed showed a significant difference between infants with and without hypocalcemia. ET via the femoral artery increases the risk of hypocalcemia. ET via the axillary artery, femoral vein and popliteal vein decreases the risk of hyperglycemia, hyponatremia and hypokalemia, respectively. Infants with higher white blood cell counts before ET have a higher risk of hypocalcemia and metabolic acidosis. A relatively lower TBIL level is significantly associated with many adverse events, such as thrombocytopenia, hypokalemia, top-up transfusion, and hypocalcemia. The relationships among the different adverse events are shown in the alluvial diagram (Fig. 1h).

**Table 2:** Comparing variables with and without adverse events during exchange transfusion

Adverse event	Variables	No	Yes	*P* value
**Hyperglycemia** **162 (86.2%)**	ABO incompatibility	15(57.7%)	50(30.9%)	0.014
	ET time	104.88 ± 20.81	95.47 ± 18.22	0.017
	ET artery axillary	5(19.2%)	5(3.1%)	0.003
	Serum potassium	3.87 ± 0.39	3.65 ± 0.53	0.046
	Cerebral hemorrhage	1(3.8%)	42(25.9%)	0.025
**Top-up transfusion after et** **95 (50.5%)**	Admission age	7.00 ± 4.04	5.85 ± 3.83	0.046
	Etiology unidentified	57(61.3%)	39(41.1%)	0.009
	IBIL at admission	431.34 ± 99.24	394.97 ± 113.56	0.021
	TBIL_bga_ at admission^a^	528.25 ± 111.54	465.65 ± 122.34	< 0.001
	TBIL_bga_ before ET	407.01 ± 107.13	370.78 ± 105.68	0.021
	Hemoglobin	142.83 ± 33.67	121.18 ± 29.40	< 0.001
**Hypocalcemia** **80 (42.6%)**	Admission age	7.51 ± 3.64	4.94 ± 3.93	< 0.001
	Breast feeding	76(70.4%)	37(46.2%)	0.001
	Formula feeding	9(8.3%)	21(26.2%)	0.002
	No feeding	0(0.0%)	7(8.8%)	0.006
	RH incompatibility	3(2.8%)	18(22.5%)	< 0.001
	G6PD deficiency	25(23.1%)	5(6.2%)	0.003
	Etiology unidentified	64(59.3%)	32(40.0%)	0.014
	ET time	99.31 ± 19.56	93.35 ± 17.32	0.032
	ET speed	5.09 ± 0.93	5.44 ± 1.02	0.014
	ET artery femoral	21(19.4%)	29(36.2)	0.016
	TBIL_bga_ at admission	528.69 ± 95.60	453.31 ± 137.64	< 0.001
	TBIL_bga_ before ET	409.50 ± 103.33	360.62 ± 107.63	0.002
	Serum bicarbonate	21.83 ± 2.60	20.64 ± 2.71	0.003
	White cell count	12.97 ± 4.77	17.01 ± 10.59	0.001
**Hyponatremia** **80 (42.6%)**	ABO incompatibility	45(41.7%)	20(25.0%)	0.026
	ET vein femoral	21(19.4%)	5(6.2%)	0.017
	Bilirubin exchange rate	0.46 ± 0.11	0.51 ± 0.08	0.004
**Thrombocytopenia** **72 (38.3%)**	Admission age	6.93 ± 4.14	5.58 ± 3.55	0.023
	TBIL at admission	453.56 ± 119.07	403.52 ± 102.60	0.004
	TBIL_bga_ Before ET	404.76 ± 109.88	362.83 ± 99.35	0.009
	Hemoglobin	137.72 ± 32.73	122.49 ± 32.29	0.002
**Metabolic acidosis** **48 (25.5%)**	Gravidity	G2(34.8%);G3(15.6%)	G2(13.3%);G3(31.1%)	0.021
	Not feeding	2 (1.4%)	5 (10.4%)	0.017
	ABO incompatibility	40(28.6%)	25(52.1%)	0.005
	Serum calcium	1.17 ± 0.12	1.13 ± 0.11	0.025
	White cell count	13.98 ± 6.92	16.75 ± 10.43	0.039
**Hypokalemia** **48 (25.5%)**	Sex (female)	50(35.7%)	26(54.2%)	0.038
	Admission age	7.00 ± 3.98	4.73 ± 3.42	0.001
	Breast feeding	91(65.0%)	22(45.8%)	0.030
	G6PD deficiency	28(20.0%)	2(4.2%)	0.018
	ET vein popliteal	20(14.5%)	0(0.0%)	0.010
	TBIL_bga_ before ET	398.06 ± 105.39	361.42 ± 110.65	0.042
	Serum calcium	1.18 ± 0.11	1.11 ± 0.14	0.001

^a^variable with subscript bga indicates that the variable was measured by a blood gas analyzer to distinguish it from the biochemistry values

Among the five machine learning models, the XGBoost model achieved the best performance in the prediction tasks of 7 adverse events (detailed information in Supplemental Tables S1 and S2). XGBoost stands for “Extreme Gradient Boosting” and is a scalable, distributed gradient-boosted decision tree (GBDT) machine learning model that solves many data science problems in a fast and accurate way [20]. XGBoost is extensively used by machine learning practitioners to create state-of-the-art data science solutions and dominates structured or tabular datasets on classification and regression predictive modeling problems.

Using the SHAP, the top 10 features (ranked from most to least important) contributing to the 7 adverse events are shown in Fig. 1a-g. Each dot in each feature corresponds to an individual case in the dataset. The position of a dot on the horizontal axis indicates the impact of the feature (SHAP value, in this study, higher SHAP values imply large contributions to adverse event risks) on model prediction, and the color of a dot reflects the feature value of the case (red for larger values, and blue for smaller). The thickness of the line comprised of individual dots is determined by the number of examples at a given value. A negative SHAP value (extending to the left) indicates reduced risk of adverse events, while a positive one (extending to the right) indicates increased risk of adverse events. Not surprisingly, all indicators directly related to adverse events (e.g., blood glucose before ET and hyperglycemia) played the most important roles in the prediction models for each adverse event. In addition to variables with statistically significant differences, a number of nonlinear relationships were identified in the interpretable AI model that will help clinical staff understand these risks in greater depth. The detailed relationships of the top 10 features identified by XAI are shown in Supplemental Figures S1-S7.

**Fig. 2:**
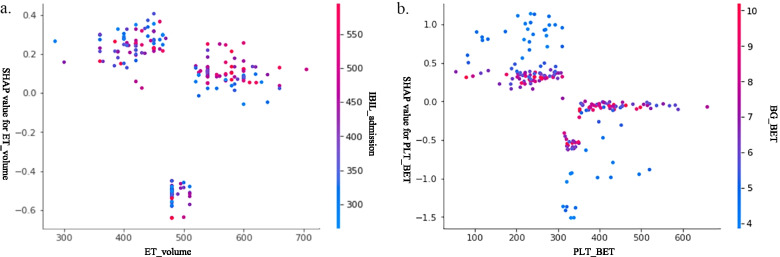
Two nonlinear risk factors for hyperglycemia during ET identified by explainable artificial intelligence. **a** ET volume with pit shape relationship; **b** low blood glucose level in infants with low platelets is a risk factor for hyperglycemia. The cases are colored according to the value of the interaction variable shown on the right side

For hyperglycemia, both the statistical analysis and XAI found that a high blood glucose level before ET, ABO incompatibility, ET time, and serum potassium are important risk factors. XAI also showed that platelet count and ET volume are associated with hyperglycemia. In Fig. 2a, b, these two variables both show a nonlinear relationship and interaction with another related variable. Both large and small ET volumes increase the risk of hyperglycemia. Only a small range of approximately 500 ml will decrease the risk. Unexpectedly, lower platelet counts, especially with lower blood glucose levels, correlate with a higher hyperglycemia risk. However, higher platelet counts with lower blood glucose levels can reduce the risk.

For top-up transfusion after ET, the statistical analysis only demonstrated that lower IBIL and TBIL contribute to this adverse event, but XAI identified that higher DBIL in infants and lower TBIL contribute to this adverse event.

**Fig. 3:**
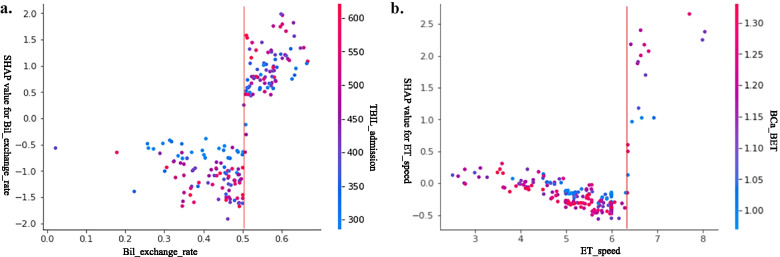
Two risk factors for which XAI provides a clear threshold (the red line) to control risk in practice. **a** A bilirubin exchange rate < 0.5 can effectively reduce the risk of hypocalcemia; **b** control of the ET speed to less than 6.3 ml/min can also reduce the risk of hypocalcemia

Although there were significant differences between different feeding modes with/without hypercalcemia, XAI did not include them as significant factors in the prediction of hypocalcemia. Traditional statistical analysis focused on the short ET time and higher ET speed, while XAI showed that a bilirubin exchange rate of ET > 0.5 was a more important risk factor for hypocalcemia, as shown in Fig. 3a. The risk of hypocalcemia decreases initially with increasing ET speed but increases significantly when the ET speed exceeds 6.3 ml/min, as shown in Fig. 3b. Both XAI and traditional analysis show that an elevated white cell count is a risk factor for hypocalcemia. XAI also identified a relationship between pH and hypercalcemia.

Except for the bilirubin exchange rate among the 3 risks identified by statistical analysis for hyponatremia, XAI showed more complex relationships between variables and hyponatremia, such as a cliff-like pattern change with a platelet count of approximately 300 × 10^9^/L. HCO_3_ also showed a reverse U-shaped relationship with hyponatremia.

Except for the lower hemoglobin identified by statistical analysis, higher serum bicarbonate, higher serum potassium, higher white cell count, and lower serum calcium all contribute to thrombocytopenia based on XAI.

**Fig. 4:**
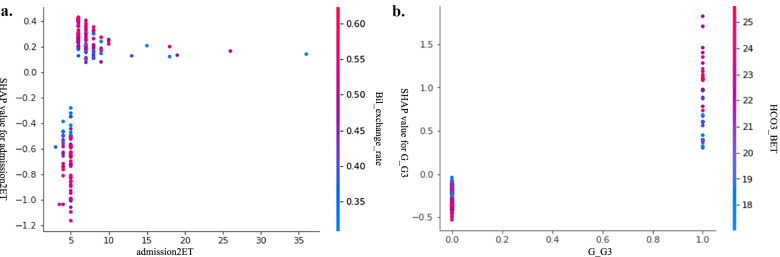
Two unexpected risk factors for metabolic acidosis. **a** Waiting time for ET seems to be associated with metabolic acidosis. **b** A third gravidity has a higher risk of coinciding with metabolic acidosis during ET

An unexpected relationship between metabolic acidosis and the waiting time until ET after admission was identified, as shown in Fig. 4a. It seems that performing ET 5 h after admission will help to control the occurrence of acidosis. A third gravidity was related to a higher risk of acidosis during ET (Fig. 4b). From the original data, there were 40% third gravidity infants vs. 11% second gravidity infants with metabolic acidosis in this cohort.

Although popliteal vein infusion was identified as a risk factor for hypokalemia in statistical analysis, the XAI considered use of the radial artery to be a more important feature. An interesting finding is that the diagnosis of bilirubin encephalopathy reduced the risk of hypokalemia. G6PD deficiency, which showed a significant difference in statistical analysis, was not among the top 10 features identified by XAI.

## Discussion

As medicine has advanced in methods to prevent erythroblastosis fetalis and phototherapy has come into widespread use, the number of ETs performed has declined [21, 22]. The consequence of this is that there is very little clinical experience in performing this procedure, despite it being an essential life-saving intervention in many emergency cases [23]. This also makes it difficult to accumulate ET cases in the clinic. The number of cases in this study (*n* = 188) is not large, but it is still the largest cohort ever published in recent years [7–10]. For machine learning models, the training sample size is crucial. In this study, the average AUC of XGBoost achieved 0.71, which is not ideal, due to the limited amount of training data. However, the results are significantly better than those of the widely used logistic regression model. We therefore believe that additional knowledge can be gained by explaining such a model in depth using SHAP.

Exchange transfusion, as a special type of blood transfusion, may have both the possible adverse events of a conventional transfusion itself and its specific adverse events, especially when performed in neonates. The results of this study reveal a high rate of adverse events associated with ET for neonatal hyperbilirubinemia. The majority of these events are asymptomatic, transient, and treatable laboratory abnormalities. It is dangerous to take these adverse events lightly since these laboratory abnormalities may cause severe complications such as cardiac arrest and convulsions [24]. The extent to which the adverse events associated with ET can be prevented is debatable [25]. This study demonstrated that explainable artificial intelligence not only achieves better performance but can also help clinicians to more deeply understand the nonlinear relationships among various clinical indicators and adverse events associated with ET.

In previous studies [7, 10], the terms “complications” and “adverse events” are often used interchangeably, which does not help the reader to clearly distinguish the difference between these two terms. Adverse events are more appropriate to describe symptoms, laboratory abnormalities, etc., that are directly caused by a particular medical intervention and service [19]. In this study, adverse events were defined by several quantitative monitored laboratory test indicators during ET. Some of the severe complications after ET, such as death, cardiorespiratory arrest, sepsis and necrotizing enterocolitis, were not defined as adverse events in this study.

In this study, we combined high-accuracy ML models and state-of-the-art local explanation methods to allow the systematic study of risks of adverse events during ET. In this study, high accuracy is necessary but insufficient; explaining models is also essential for drawing hypotheses. XAI has repeatedly identified a number of risk factors that have also been identified through traditional statistical models [7–10]. However, some factors did not show significant differences in traditional statistical analysis, such as the XAI-identified elevation of direct bilirubin increasing the risk of top-up transfusion after ET. This result was also supported by a basic study that showed that direct bilirubin triggers anemia [26]. Since direct bilirubin and total bilirubin have opposite effects on the risk of top-up transfusion, the ratio of direct bilirubin to total bilirubin could be used as a more significant indicator of the associated risk.

A clear threshold rather than a traditional correlation is more conducive to gaining actionable clinical knowledge to effectively control risk. XAI, which can present a clear threshold, as shown in Fig. 3, offers another advantage of using it to analyze clinical data. The knowledge of controlling the ET speed not to exceed 6.3 ml/min is clear, unambiguous and actionable for clinicians, as opposed to the traditional analysis of just one correlation coefficient.

XAI could identify some novel relationships that could be missed by traditional statistical analysis due to nonlinear relationships or simple distribution imbalance issues. Such novel relationships should inspire researchers to study them further. Many of these relationships are shown in the supplemental figures.

There are several limitations to our study. First, only a limited number of cases were used to build the AI models, and the overall performance of the model is not very high. As such, external data training and validation are required in the future. Fortunately, the XGBoost model chosen for this study has been shown in previous evaluations to achieve good prediction results with a small sample size [27]. Second, the relationships and interactions detected by XAI cannot be claimed to be causal. The novel relationships discovered should be validated in more strictly designed causal inference studies. Third, this study did not include severe adverse events and permanent serious sequelae due to their rarity in current advanced neonatal care and the training requirements of machine learning models.

In conclusion, we used traditional statistical analysis and XAI to identify the risk factors for 7 major adverse events during exchange transfusion in neonatal hyperbilirubinemia. The XAI model achieved better performance in predicting adverse events and provided more useful and actional knowledge for clinicians.

## Supplementary Information

**Additional file 1.** Supplemental material.

## Data Availability

All data generated or analyzed during this study are included in this article and its supplementary material files. Further enquiries can be directed to the corresponding author.

## Abbreviations

ET: Exchange Transfusion
XAI: Explainable Artificial Intelligence
SHAP: SHapley Additive exPlanations
TBIL: Total Bilirubin
IBIL: Indirect Bilirubin
DBIL: Direct Bilirubin
G6PD: Glucose-6-Phosphate Dehydrogenase
