# Supplementary material for: Understanding the risk factors for adverse events during exchange transfusion in neonatal hyperbilirubinemia using explainable artificial intelligence
Source: BMC Pediatr. 2022 Sep 30;22:567. doi: 10.1186/s12887-022-03615-5 (PMC9523933; doi:10.1186/s12887-022-03615-5)
Supplement: Supplementary file 1 — Additional file 1. Supplemental material. [file 12887_2022_3615_MOESM1_ESM.docx]

Supplemental material

Table S1 All the variables used to build machine learning model and their abbreviation

| Variable class | Variable abbreviation | Variable explain |
| --- | --- | --- |
| Sex | Sex_1 | Sex changed into TURE/FALSE in two variables in the model |
|  | Sex_2 |  |
| Gravidity | G_G1 | Gravidity changed into TRUE/FALSE for 7 variables |
|  | G_G2 |  |
|  | G_G3 |  |
|  | G_G4 |  |
|  | G_G5 |  |
|  | G_G6 |  |
|  | G_G7 |  |
| Parity | P_P1 | Parity changed into TRUE/FALSE for 3 variables |
|  | P_P2 |  |
|  | P_P3 |  |
| Gestational age | Gestational_age | Gestational age (weeks) |
| Delivery mode | Birth_way | 1for normal natural delivery; 2 for C-section |
| Apgar score | Apgar | Apgar score at birth |
| Birth weight | Birth_weight | Birth weight (g) |
| Weight at admission | admission_weight | Weight when admission |
| Age at admission | admission_age | Age when admission in days |
| Feeding type | Feeding_breast | Breast feeding |
|  | Feeding_formula | Formula feeding |
|  | Feeding_none | Not start feeding |
|  | Feeding_mixed | Formula and breast mixed feeding |
| Diagnose | Diag_ABO_hemolytic | ABO hemolytic / ABO incompatibility |
|  | Diag_RH_hemolytic | RH hemolytic/ RH incompatibility |
|  | Diag_BE | Bilirubin encephalopathy |
|  | Diag_anemia | anemia |
|  | Diag_premature | premature |
|  | Diag_sepsis | Sepsis |
|  | Diag_G6PD | G6PD deficiency |
|  | Diag_NEC | Necrotizing enterocolitis |
|  | Diag_PM | Purulent meningitis |
|  | Diag_HB | Hyperbilirabinemia without etiology |
| ET waiting time | Admission2ET | The time from admission to ET in hours |
| ET volume | ET_volume | ET volume (ml) |
| ET time | ET_time | ET length of time (minutes) |
| ET speed | et_speed | Calculate average speed by ET_volume/ET_time |
| ET artery | ET_artery_radial | Radial artery |
|  | ET_artery_brachial | Brachial artery |
|  | ET_artery_femoral | Femoral artery |
|  | ET_artery_axillary | Axillary artery |
|  | ET_artery_scalp | Scalp |
|  | ET_artery_ulnar | Ulnar artery |
| ET vein | ET_vein_axillary | Axillary vein |
|  | ET_vein_basilic | Basilic vein |
|  | ET_vein_mediancubital | Mediancubital vein |
|  | ET_vein_hand | Hand dorsal vein |
|  | ET_vein_femoral | Femoral vein |
|  | ET_vein_popliteal | Popliteal vein |
|  | ET_vein_foot | Foot dorsal vein |
| Baby blood group | Baby_RH_positive | The RH、ABO blood type of baby were changed into TRUE/FALSE value in the model. |
|  | Baby_RH_negative |  |
|  | Baby_ABO_A |  |
|  | Baby_ABO_B |  |
|  | Baby_ABO_AB |  |
|  | Baby_ABO_O |  |
| Father blood group | Father_RH_positive | The RH、ABO blood type of baby’s father were changed into TRUE/FALSE value in the model. |
|  | Father_RH_negative |  |
|  | Father_ABO_A |  |
|  | Father_ABO_B |  |
|  | Father_ABO_AB |  |
|  | Father_ABO_O |  |
| Mother blood group | Mother_RH_positive | The RH、ABO blood type of baby’s mother were changed into TRUE/FALSE value in the model. |
|  | Mother_RH_negative |  |
|  | Mother_ABO_A |  |
|  | Mother_ABO_B |  |
|  | Mother_ABO_AB |  |
|  | Mother_ABO_O |  |
| TBIL at admission | TBIL_admission | Total bilirubin at admission measured by biochemistry |
| TBIL after ET | TBIL_ET | Total bilirubin after ET measured by biochemistry |
| IBIL at admission | IBIL_admission | Indirect bilirubin at admission measured by biochemistry |
| IBIL after ET | IBIL_ET | Indirect bilirubin after ET measured by biochemistry |
| DBIL at admission | DBIL_admission | Direct bilirubin at admission measured by biochemistry |
| DBIL after ET | DBIL_ET | Direct bilirubin after ET measured by biochemistry |
| TBIL_bga_ before ET | BG_TBIL_BET | Total bilirubin before ET measured by blood gas analyzer |
| TBIL_bga_ during ET | BG_TBIL_IET | Total bilirubin during ET (at time about half of volume exchanged ) measured by blood gas analyzer |
| TBIL_bga_ after ET | BG_TBIL_AET | Total bilirubin after ET measured by blood gas analyzer |
| Bilirubin exchange rate | Bil_exchange_rate | Calculate by (BG_TBIL_BET – BG_TBIL_AET)/BG_TBIL_BET |
| pH | PH_BET | pH level before ET |
| Serum bicarbonate | HCO3_BET | Serum bicarbonate before ET |
| Serum glucose | BG_BET | Serum glucose before ET |
| Serum potassium | BK_BET | Serum potassium before ET |
| Serum calcium | BCa_BET | Serum calcium before ET |
| Serum sodium | Bna_BET | Serum sodium before ET |
| Hemoglobin | HGB_BET | Hemoglobin before ET |
| White cell count | WC_BET | White cell count before ET |
| Platelet count | PLT_BET | Platelet count before ET |

Table S2 The AUC performance of 5 models in prediction 7 adverse events during exchange transfusion.

|  | hypokalemia | hyperglycemia | Top-up transfusion | hypocalcemia | hyponatremia | thrombocytopenia | acidosis | Average in 7 tasks |
| --- | --- | --- | --- | --- | --- | --- | --- | --- |
| XGB | 0.761 | **0.750** | **0.646** | **0.823** | **0.616** | **0.708** | **0.697** | **0.714429** |
| Logistic Regression | 0.526 | 0.303 | 0.664 | 0.700 | 0.530 | 0.700 | 0.402 | 0.546429 |
| GaussianNB | 0.532 | 0.440 | 0.561 | 0.712 | 0.576 | 0.490 | 0.573 | 0.554857 |
| Kneighbors | 0.518 | 0.433 | 0.421 | 0.674 | 0.486 | 0.603 | 0.425 | 0.508571 |
| RandomForest | **0.802** | 0.659 | 0.537 | 0.811 | 0.601 | 0.629 | 0.691 | 0.675714 |
| Average of 5 models | 0.6278 | 0.517 | 0.5658 | 0.744 | 0.5618 | 0.626 | 0.5576 | 0.6 |


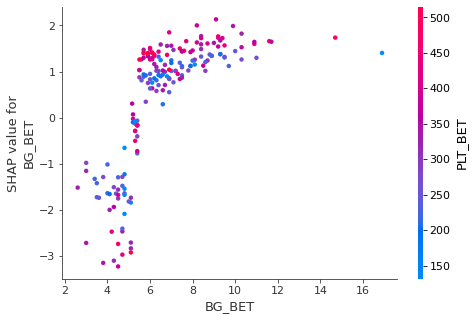

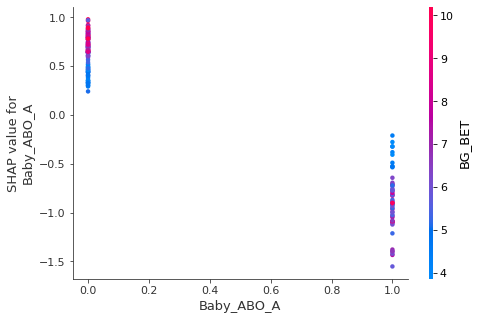

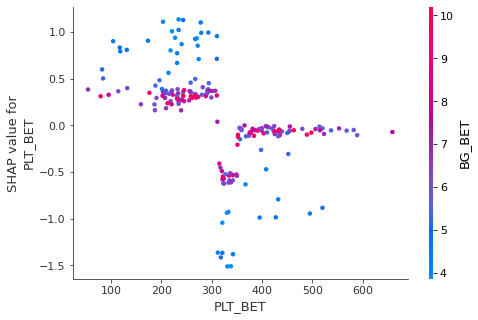

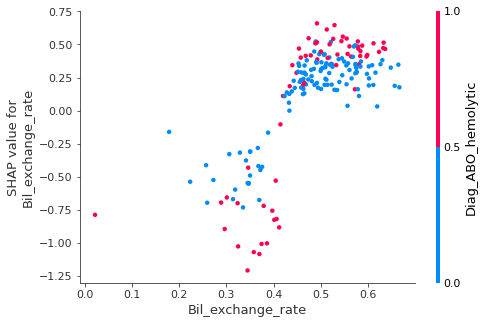

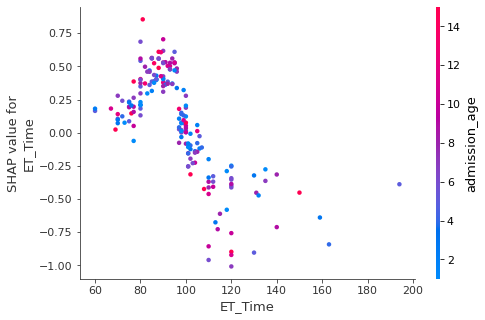

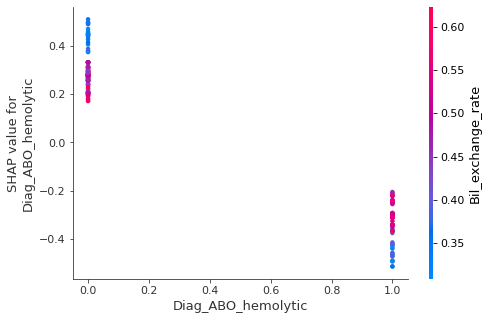

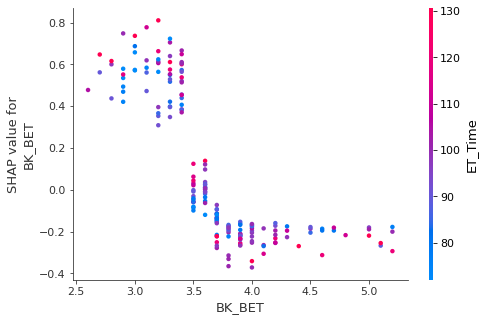

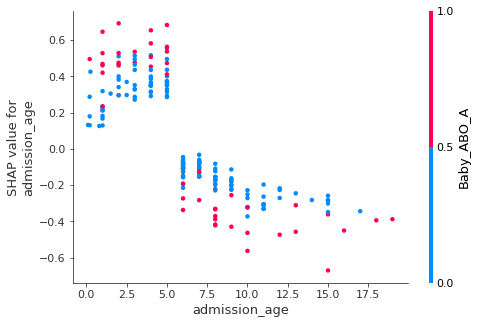


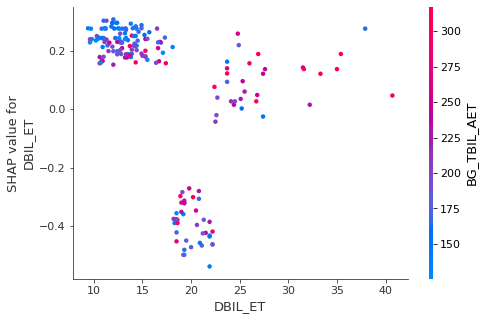

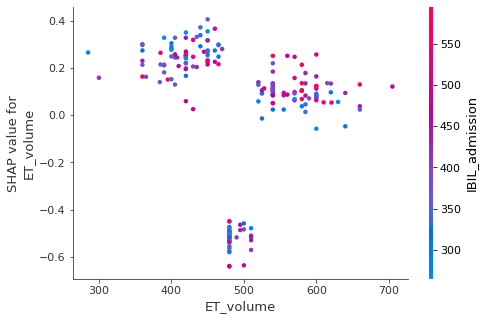


Figure S1 Top 10 important factors associated with hyperglycemia during ET


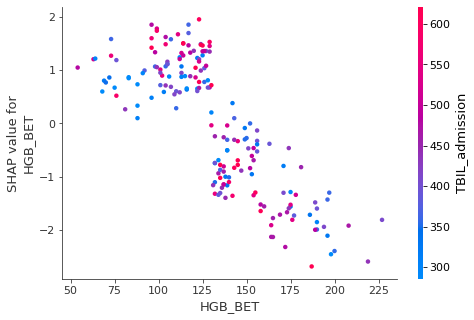

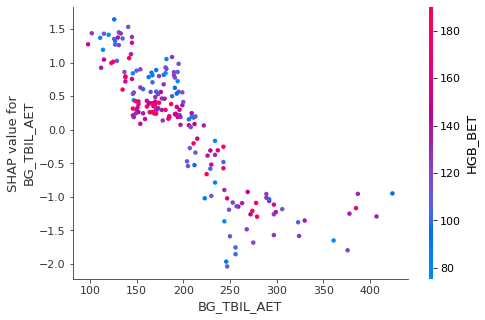

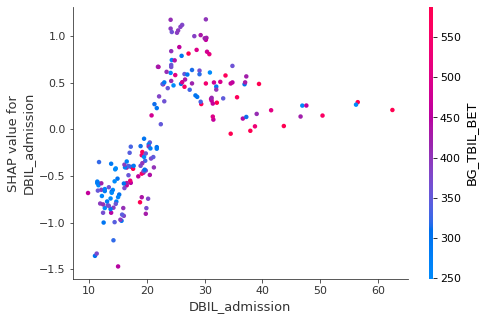

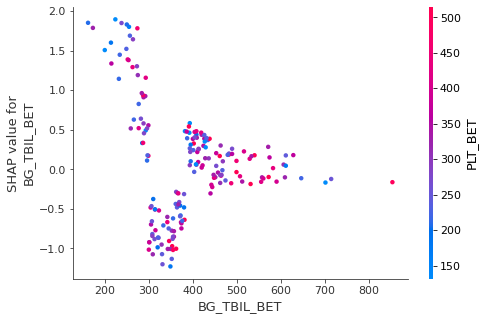

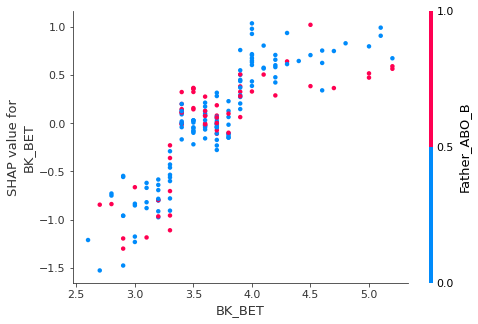

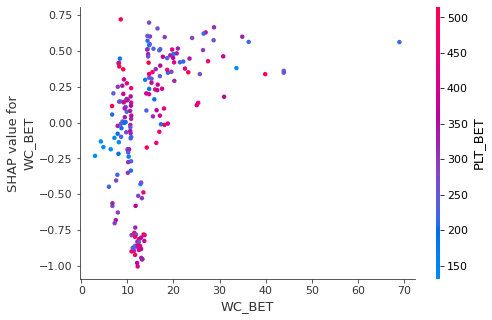

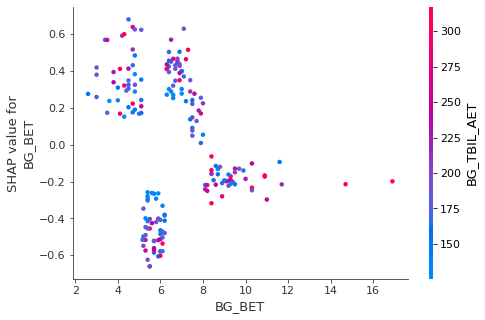

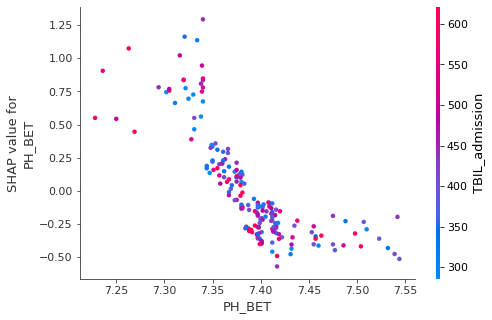

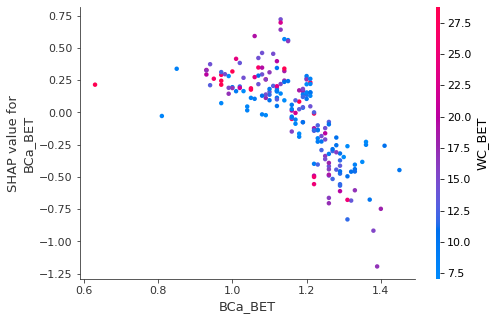

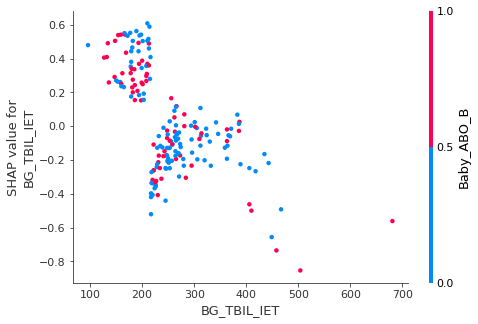


Figure S2 Top 10 important factors associated with top-up transfusion after ET


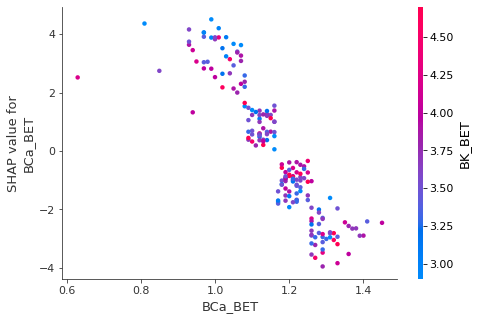

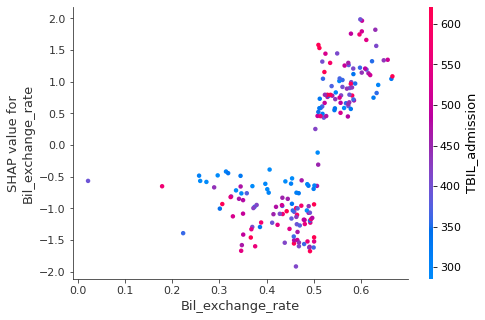

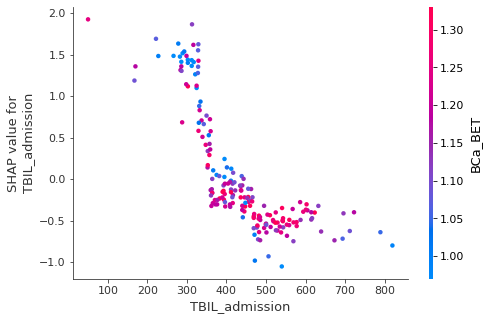

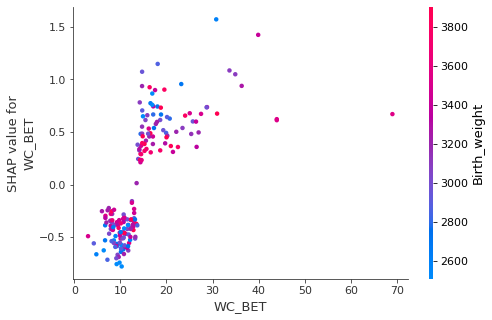

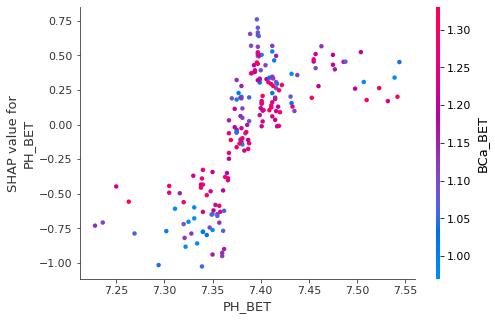

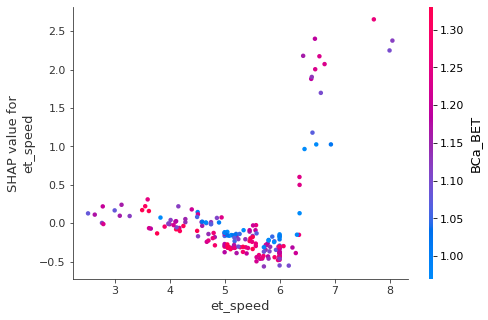

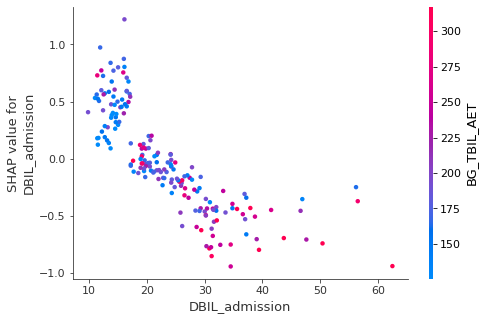

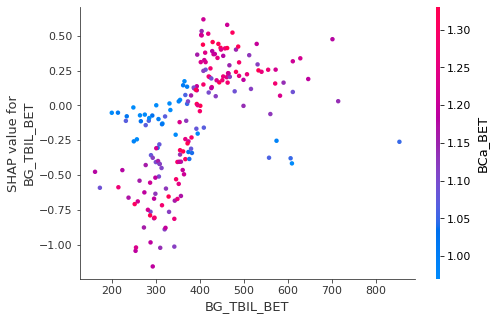

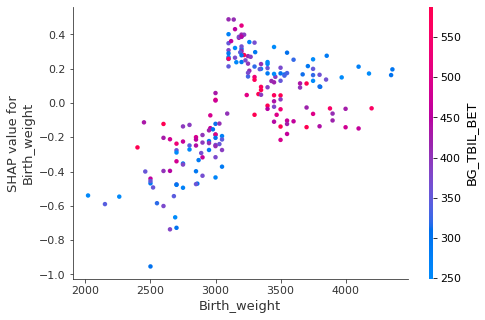

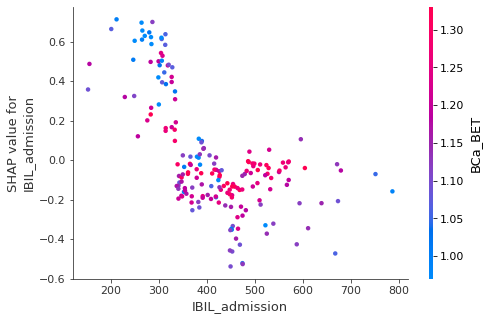


Figure S3 Top 10 important factors associated with hypercalcemia during ET


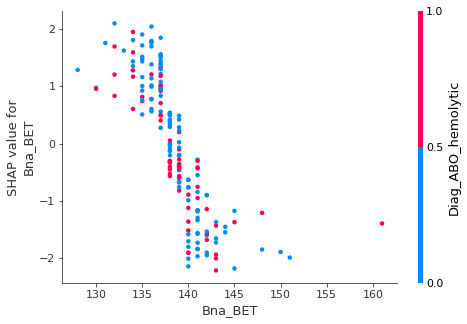

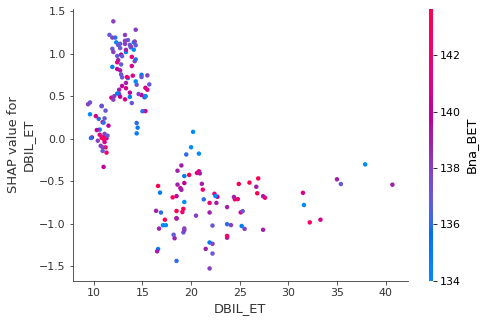

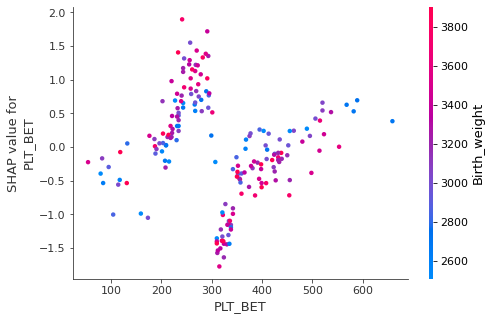

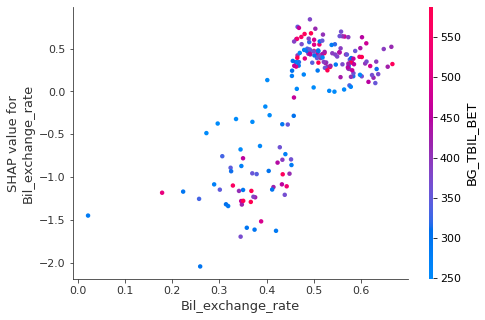

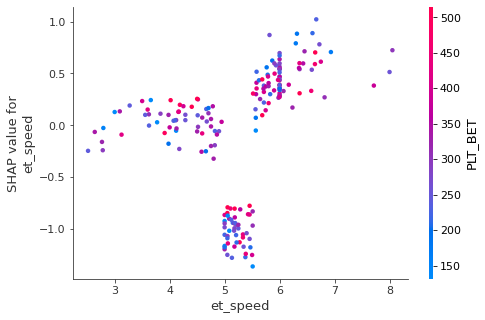

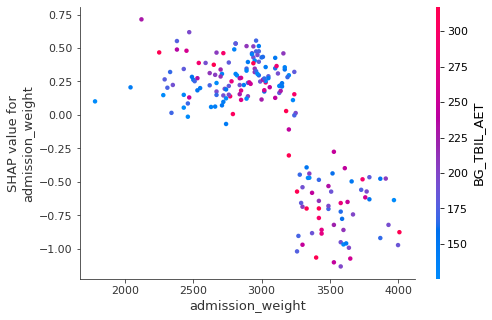

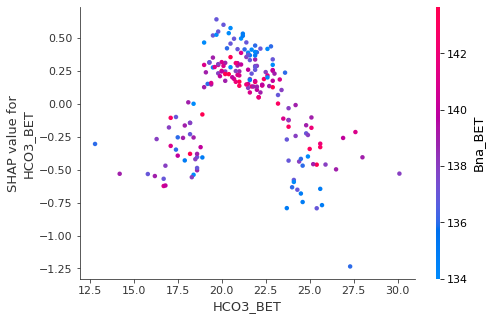

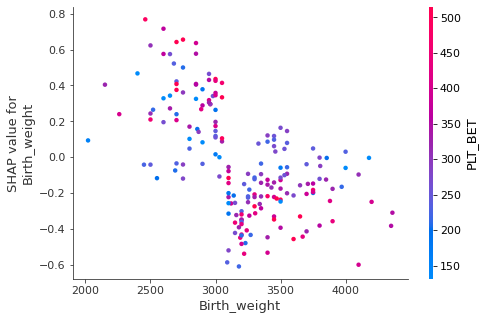

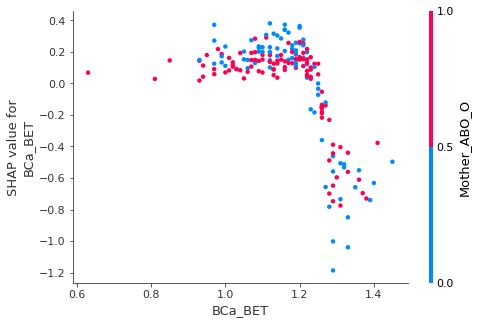

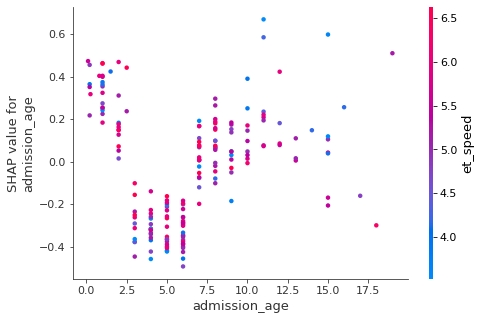


Figure S4 Top 10 important factors associated with hyponatremia during ET


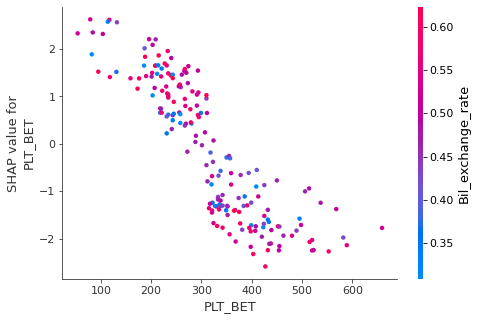

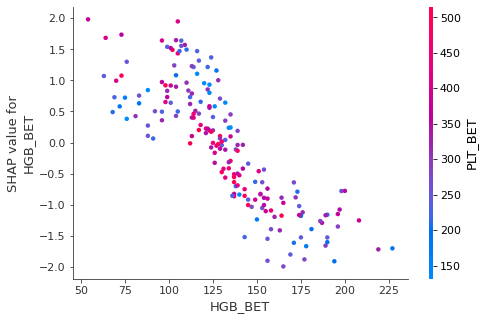

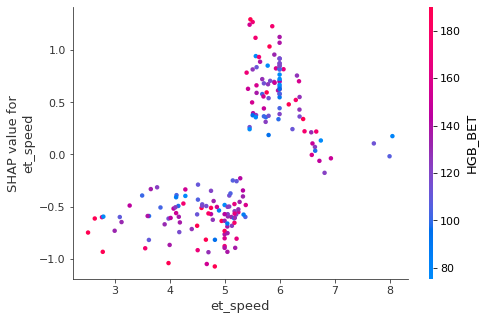

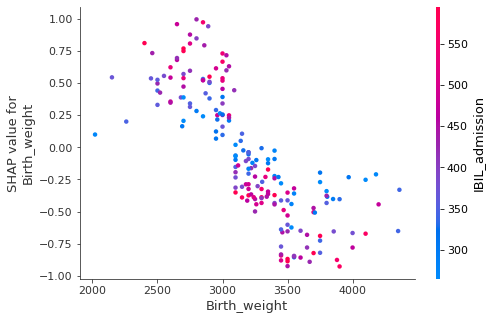

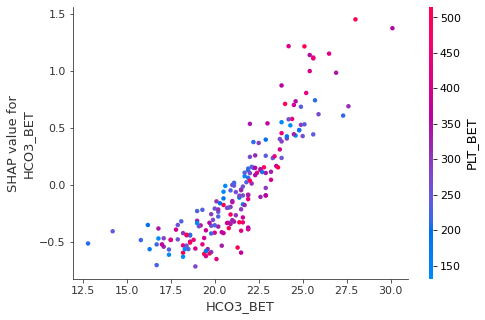

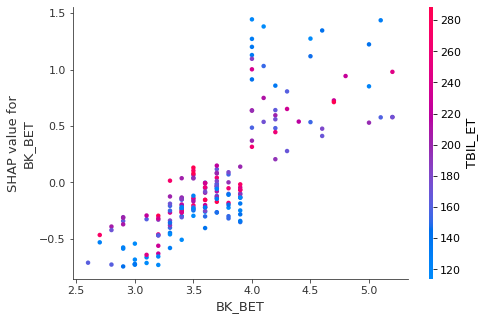

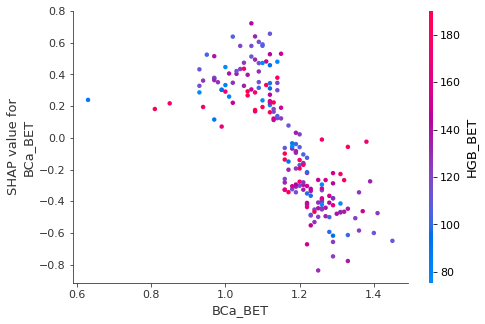

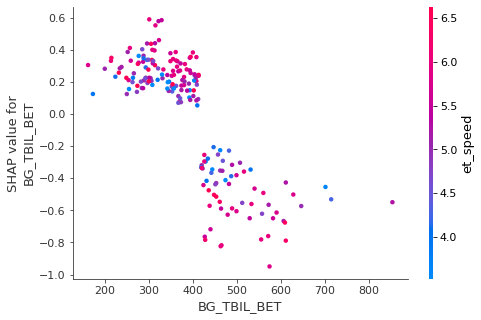

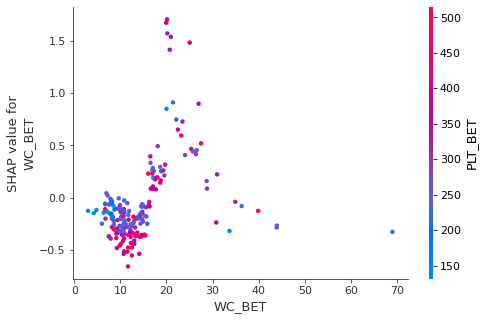

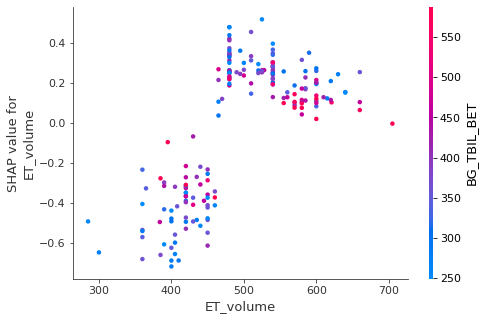


Figure S5 Top 10 important factors associated with thrombocytopenia during ET


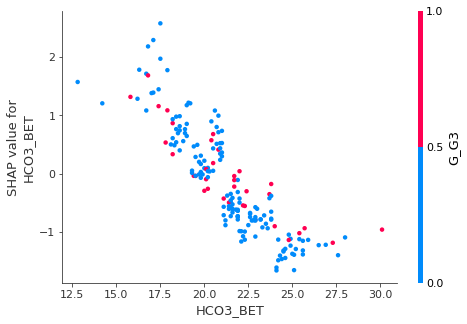

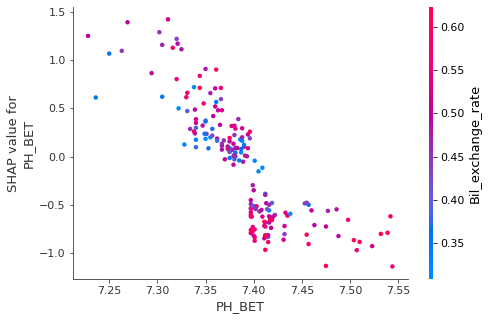

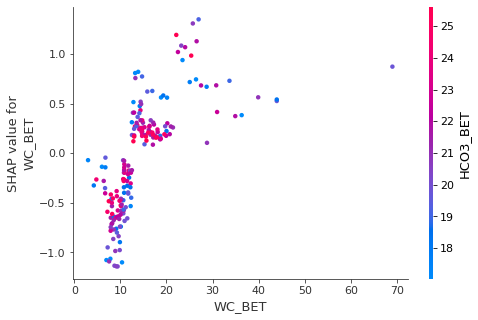

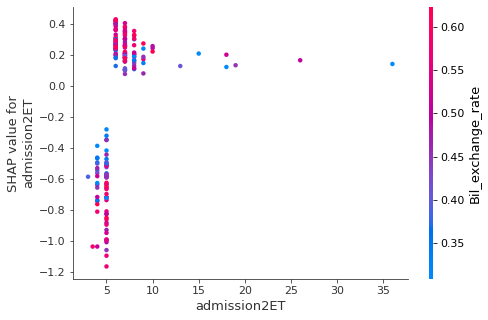

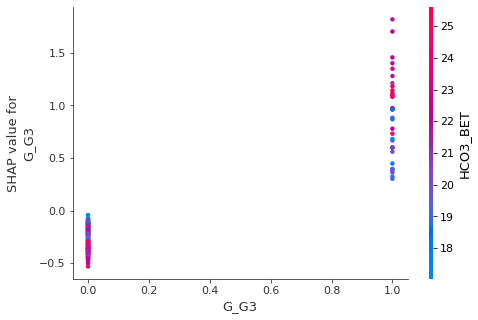

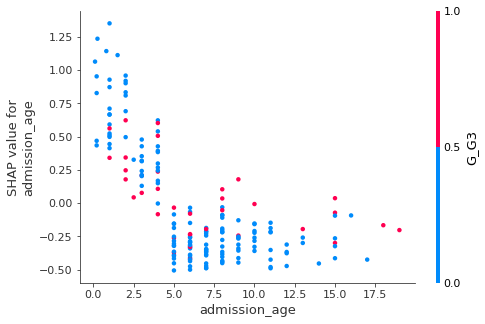

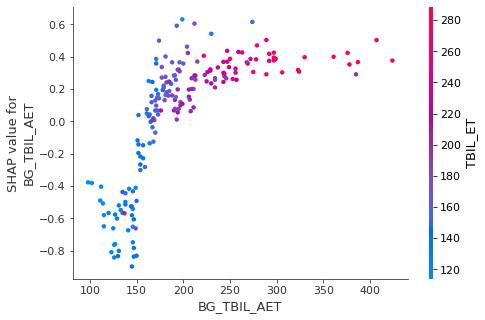

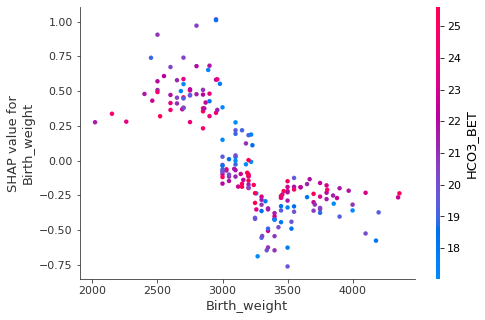

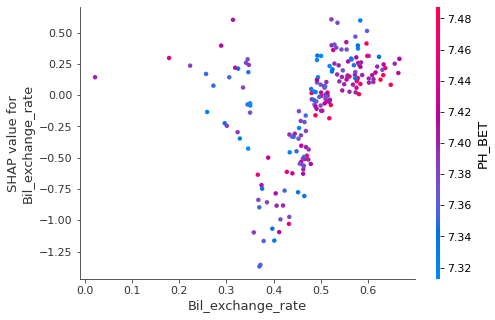

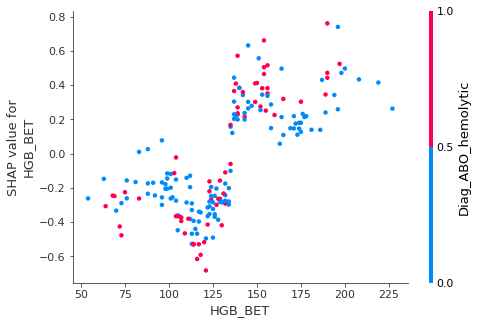


Figure S6 Top 10 important factors associated with metabolic acidosis during ET


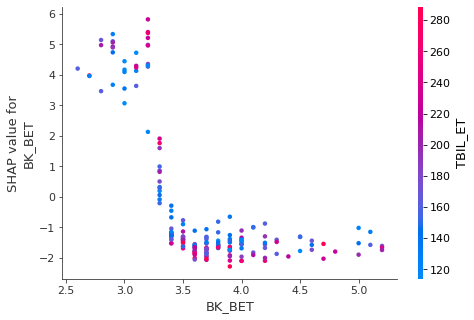

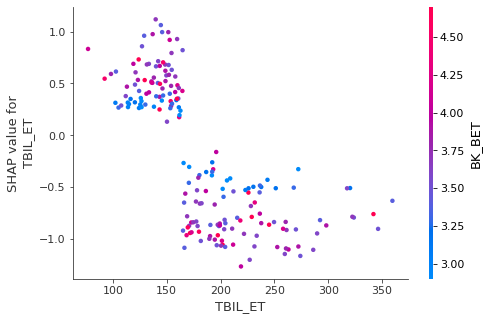

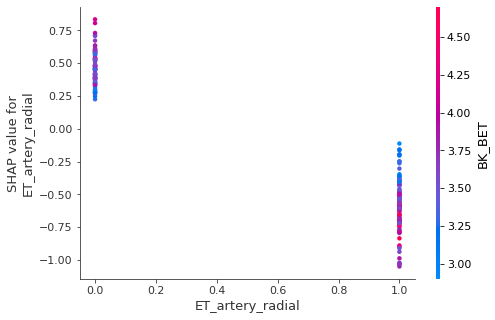

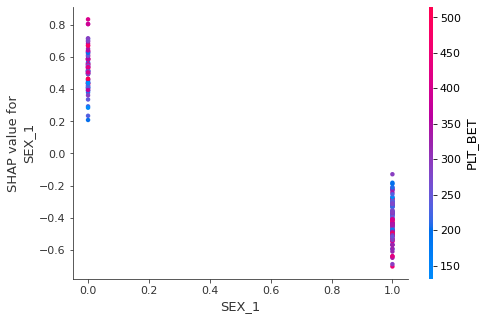

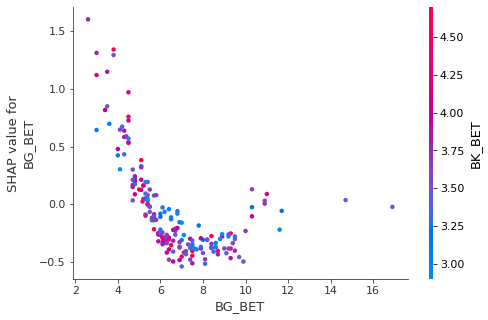

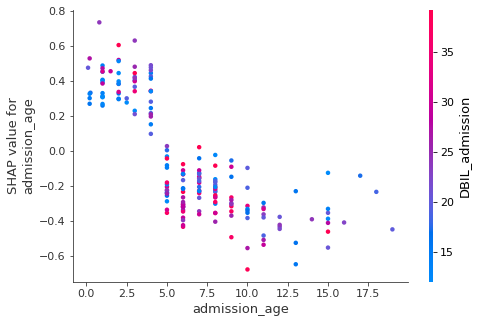

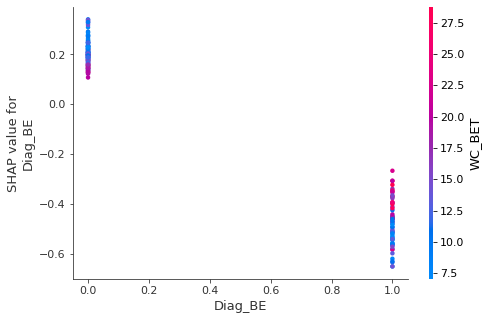

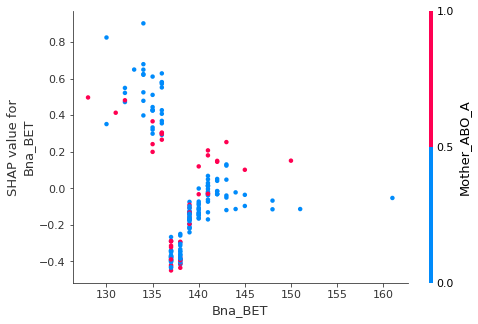

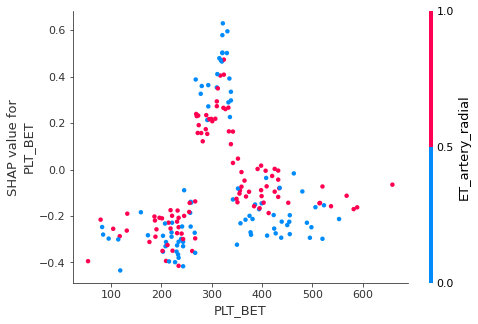

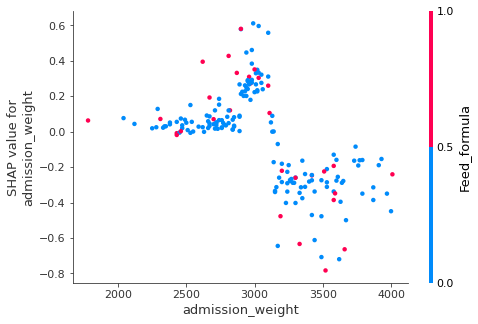


Figure S7 Top 10 important factors associated with hypokalemia during ET
